# Navigating Complexity: A Case Report on Interplay of Intellectual Disability (ID), Schizophrenia, Clozapine Treatment and Chemotherapy

**DOI:** 10.1192/bjo.2024.674

**Published:** 2024-08-01

**Authors:** Alexandra Marconi, Alaa Martin

**Affiliations:** 1 Essex Partnership University Foundation Trust, Chelmsford, United Kingdom; 2 Essex Learning Disability Partnership, Colchester, United Kingdom

## Abstract

**Aims:**

The use of Clozapine treatment requires rigorous and mandatory monitoring due to the side effects profile of Clozapine. Certain clinical situations may pose a dilemma for clinicians such as concomitant use of clozapine during myelosuppressive chemotherapy. There is limited evidence-based data regarding Clozapine and chemotherapy. We report on a case of a clozapine-stabilized, Schizophrenia patient with Mild ID who was diagnosed with High Grade B-cell Non-Hodgkin's Lymphoma (NHL) requiring chemotherapy. The challenges of this complex case are detailed in this paper.

**Methods:**

A 56-year old man with a diagnosis of Mild ID, Schizophrenia and OCD. The patient has been taking Clozapine since 2001 daily dose of 600-400 mg for the past 20 years. Unfortunately, he was diagnosed with High Grade NHL in 2023. The decision was reached to continue Clozapine while undergoing chemotherapy sessions with frequent blood monitoring. Towards the end of his chemotherapy his bloods showed dangerously low (Clozapine red alert) requiring stopping Clozapine. The patient started showing signs of relapse in his mental state and subsequently commenced on Olanzapine. He continued to show signs of relapse and didn't recover to his previous baseline; the treatment plan is adding another antipsychotic or considering re challenging Clozapine.

**Results:**

This report contributes to a very limited literature on the concurrent use of clozapine with chemotherapy and the use of Clozapine “*outside license*”. The main treatment options facing clinician is stopping or continuing clozapine during chemotherapy. The dilemma of taking the path of withdrawing a medication on which a patient is stabilized may compromise psychiatric stability, yet there is a valid argument that such inconvenience would present more favourable outcome than facing the serious haematological risks of neutropenia. There is a need for robust and close liaison between psychiatrists, oncologist, and haematologist on the various clinical considerations.

**Conclusion:**

In summary, both clozapine and chemotherapy are known to cause neutropenia and agranulocytosis. The clinical decision to continue clozapine during chemotherapy could be challenging. Clinicians should be aware that psychotic decompensation in such patients would inevitably increase morbidity and perhaps mortality due to nonadherence to all proposed treatment, including chemotherapy. In the absence of guidelines and given the nature of treatment-resistant symptoms, clinicians should work in a multidisciplinary approach and carefully weigh the risks and benefits of continuing clozapine during chemotherapy.

